# The intra-class heterogeneity of immunophenotyping and immune landscape in oesophageal cancer and clinical implications

**DOI:** 10.1080/07853890.2021.1912385

**Published:** 2021-04-16

**Authors:** Yujie Xie, Xiaoshun Shi, Ying Chen, Bomeng Wu, Xiaolin Gong, Weicheng Lu, Wanli Lin

**Affiliations:** a Department of Thoracic Surgery, The People’s Hospital of Gaozhou, Gaozhou, China; b Guangdong Esophageal Cancer Institute, Guangzhou, China; c Department of Thoracic Surgery, Nanfang Hospital, Southern Medical University, Guangzhou, China; d Department of Graduate School, Guangdong Medical University, Zhanjiang, China

**Keywords:** Immunophenotyping, immune landscape, oesophageal cancer tumour microenvironment, immunotherapy

## Abstract

**Background:**

The response rate and survival benefit of immunotherapy vary among patients, implying specific immune status of an individual could be associated with the effect of immunotherapy. However, in-depth studies of immune subtypes (ISs), immune landscape and tumour microenvironment of oesophageal cancer (ESCA) and their clinical implications are less reported.

**Methods:**

We first accessed data from publicly available databases and preprocessed it based on a standard protocol. Then, ISs were identified by unsupervised learning. Thereafter, the association of these ISs and tumour mutation burden (TMB), biomarkers of chemotherapy-induced immune response, tumour markers were also assessed. In addition, the immune characteristics, immune landscape, co-expression network of immune genes, and clinical implications were visualized and analysed.

**Results:**

We identified three immunoclusters based on immune-associated genes with intra-class heterogeneity and prognostic value. Cluster-specific associations with TMB, markers of chemotherapy-induced immune response, and tumour markers were revealed. A 4-gene signature (risk score= −0.16514291×*BHLHE22*−0.03964046×*MXRA8*−0.15242778×*SLIT2*−0.05553572×*SPON1*) based on co-expressed genes in the immunoclusters was developed and externally validated.

**Conclusions:**

In summary, we identified clinically relevant immunoclusters in both adenocarcinoma and squamous cell carcinoma of oesophagus, revealing the necessity of assessing the complexity and diversity of immune microenvironment for cancer immunotherapy.

## Background

Oesophageal cancer (EC) is a common malignant tumour in the digestive system, ranking sixth and seventh cancer-related deaths in the world [1]. There are two main histological subtypes of EC, including oesophageal adenocarcinoma (EAC) and oesophageal squamous cell carcinoma (ESCC). EAC incidence is mainly in Caucasian [2], while ESCC is more common in developing countries, such as China [3]. Surgery, chemotherapy and radiotherapy alone or in combination are treatments for EC. However, the estimated 5-year survival rate of EC ranges from 10 to 39.7% [3–7]. In recent years, immunotherapy for EC has been reported [8,9], and clinical trials are also underway, providing novel strategies for improving patient’s survival with EC. Therefore, the identification of immune subtypes (ISs) responsible for tumour immune microenvironment (TIME) of EC is crucial for a better understanding of a patient’s immune status, which is associated with the effect of immunotherapy, treatment decision and prognosis prediction of EC.

Tumour microenvironment (TME) refers to the various surrounding microenvironment in which tumour cells exist, including tumour-nourishing blood vessels, numerous signalling molecules and complex extracellular matrix (ECM) components [10]. Among them, the composition of immune cells in tumours and the complexity and diversity of the immune environment created by them constitute the TIME, affecting the growth and development of cancer cells. The TIME's tumour suppressors are mainly conducted by cytotoxic T cells (CTL) and natural killer (NK) cells, usually with a decrease in number. The cells act as tumour promoters by immunosuppressive function include myeloid-derived suppressor cells (MDSCs), regulatory T cells (Tregs) and tumour-associated macrophages (TAMs), which are the targets of immunotherapy in recent years [11]. Therefore, revealing the association of tumour IS with immune cells is helpful to understand the role of TIME in different cancer subtypes. In addition, the immune landscape analysis based on the characteristics of tumour immune cells is a novel approach to evaluate TIME, but the immune landscape of oesophagus cancer is unclear. In sum, the associations of TIME with tumour gene expression and mutation, as well as the impacts of TIME on primary tumours, chemotherapy, targeted therapy and immunotherapy, need further investigations.

Due to few reports on immunophenotyping of EC, this study aimed to identify ISs in EC with clinical value and its association with tumour mutation burdens (TMBs), clinical tumour biomarkers, tumour immune gene expression, infiltrative immune cell composition and potential immune function (activation and suppression). By providing an IS with predictive prognosis value based on TIME in EC, our ISs may also hint at the direction of improving EC therapeutic effect for immunotherapy and reasonable combination strategies.

## Materials and methods

### Data accession and preprocessing

We used the TCGA Genomic Data Commons (https://portal.gdc.cancer.gov/) to download the RNA-Seq data of TCGA-ESCA. After preprocessing, 161 samples with RNA-Seq data were obtained. The GEO data were downloaded from Gene Expression Omnibus (https://www.ncbi.nlm.nih.gov/geo/query/acc.cgi?acc=GSE53624), accessing the GSE53624 microarray data with survival information with a total of 119 samples [12]. The data preprocessing procedure of TCGA-ESCA were the removal of (1) normal tissue data, (2) samples with no survival information, (3) genes with expression level (TPM) equal to 0 in more than 50% of the samples and (4) log2 (TPM + 1) transformation. In terms of GEO data preprocessing, removal of data from normal tissue and without survival data was performed. The mutation data set of TCGA-ESCA was downloaded and processed by the mutect2 tool, then the patient's TMB was calculated.

### Identification of immune related genes

Immune-related genes were selected based on (1) immune cell-specific genes derived from single-cell RNA-Seq data; (2) genes encoded co-stimulatory and co-suppressive molecules; (3) genes encoded cytokines and cytokine receptors; (4) genes involved in antigen processing and presentation. The expression profiles of these genes were retrieved from TCGA-ESCA and GSE53624 dataset.

### Identification of immune subtypes and immune gene modules

We applied consensus clustering by ConsensusClusterPlus to classify the samples [13]. The ISs based on 1951 immune-related gene expression were obtained [14]. We used the PAM algorithm and the Spearman correlation of 1 as a measurement of distance, and then each bootstraps process included 80% of the training set patients for 500 times were performed. The optimal classification was determined by calculating the consistent matrix and the cumulative distribution function (CDF) estimator. The immune gene modules were also identified based on the same setting.

### Functional analysis of the immune gene modules

We annotated the biological functions of immune gene modules by DAVID (v6.8) tool and annotated the biological processes of the genes in each module by Gene Ontology. We applied the ANOVA algorithm to evaluate the association between ISs and 57 previously reported immune-related molecular and cellular characteristics [15].

### The association of immune subtypes with clinical, molecular and cellular characteristics

By using the age, gender, T stage, N stage, M stage, TNM stage and grade of differentiation as the covariates in training set samples against the overall survival (OS) rate as the endpoint, we applied log-rank test, univariate and multivariate Cox regression method to evaluate the prognostic value of ISs. Then in the validation set, the variance analysis is used to evaluate the correlation between ISs and various immune-related molecular and cellular characteristics. The tumour immune dysfunction and exclusion (TIDE) algorithms were used to predict TCGA-ESCA patients' response to immune checkpoint inhibitors [16].

### The immune landscape of oesophagus

Taking the dynamic characteristics of the immune system into account, we used graph-based learning methods for dimensionality reduction analysis. In this study, we extended this method, which is previously used to simulate cancer progression and to define developmental trajectory analysis for single-cell gene expression data, to study immune gene expression profiles [17,18]. This immune landscape analysis reflects the relationship between patients in a nonlinear manner, which may complement the discrete ISs defined in the linear Euclidean space.

### Construction of immune modules based immune gene co-expression

Weighted gene co-expression network analysis [19] was used to identify immune modules potentially associated with clinical features. By setting a soft threshold power (*β*) set at 10 and correlation coefficient *R*-squared value >0.85, we applied the topological overlap matrix method [20] to convert data into a weighted adjacency matrix. Under dynamic tree cutting with genes in each module no less than 40, the eigengenes value is used to cluster the modules. Similar modules were merged into a new module by height = 0.25, deepSplit = 4 and minModuleSize = 40.

### Development and validation of immune gene risk score

Genes whose correlation coefficient greater than 0.75 in the significant modules were subjected to univariate Cox proportional hazard regression analysis, with *p*<.05 as the threshold for gene filtration. Then, we calculated the risk score of each sample according to the expression level of the sample, with *Z*-score normalization. The high-risk group was defined as risk score greater than zero and low-risk group as that less than zero, and then the Kaplan–Meier curve was used to visualize survival outcome.

### Statistical analysis

Data processing, visualization and statistical analysis were done by R 3.6.1 (R Foundation for Statistical Computing, Vienna, Austria). We used one-way ANOVA or the Kruskal–Wallis test to compare differences among different groups, and the Kaplan–Meier analysis was employed to analyse survival data. In brief, we considered two-sided *p*<.05 as statistical significance.

## Results

### The immune subtypes of oesophagus cancer

**Figure 1.**
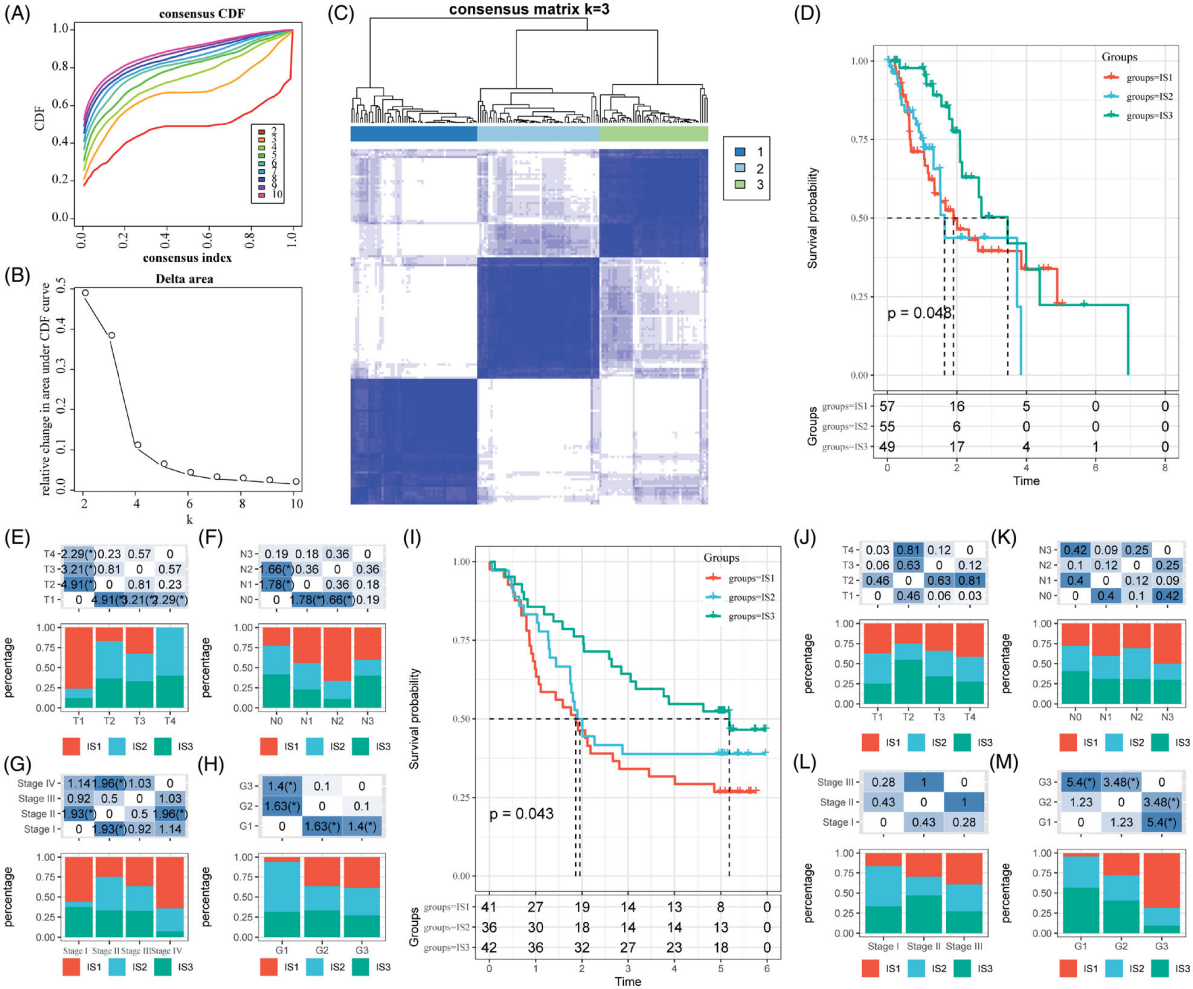
The immune subtypes in oesophageal cancer. (A) The CDF curve in TCGA-ESCA cohort samples. (B) The CDF Delta area curve of TCGA-ESCA cohort sample. (C) The clustering heat map when consensus *k* = 3. (D) The prognosis value of the three subtypes visualized by *K*–*M* curve in TCGA-ESCA cohort. The distribution of the three immune subtypes based on T stage (E), N stage (F), TNM stage (G) and tumour grade (H) in TCGA-ESCA cohort. (I) The prognosis value of the three subtypes visualized by *K*–*M* curve in GSE63524 cohort. The distribution of the three immune subtypes based on T stage (J), N stage (K), TNM stage (L) and tumour grade (M) in GSE63524 cohort. The statistical significance of the distribution difference between groups are calculated by the –log10 (*p* value).

According to the Consensus clustering CDF, it can be observed that clustering reached a stable status when the *k* equals 3 (Figure 1(A,B)), resulting in three ISs (Figure 1(C)). Further prognostic analysis based on these three ISs, significant prognostic differences among groups are revealed in Figure 1(D). Overall, IS3 subtypes have a better prognosis, while IS1 subtypes are worse than others. In addition, by comparing the relationship among these three molecular subtypes and age, gender, T stage, N stage, M stage, TNM stage and grade, we observed that there are significant distributions in T stage, N stage, TNM stage and grade differentiation, as shown in Figure 1(E–H). As shown in Figure S1A–C, there are no significant distributions among the three ISs regarding M stage, age and gender. For external validation, the same bioinformatic analysis for molecular typing on the GSE53624 microarray data was performed. Consistently, the three ISs shared similar survival curves with better prognosis in IS3 (Figure 1(I)). Tumour grades are significantly distributed in the three ISs, while there is no significant difference in terms of age, gender, T stage, N stage and TNM stage (Figure S1D,E and Figure 1(J–L)).

### Differences of TMB distribution in the immune subtypes

**Figure 2.**
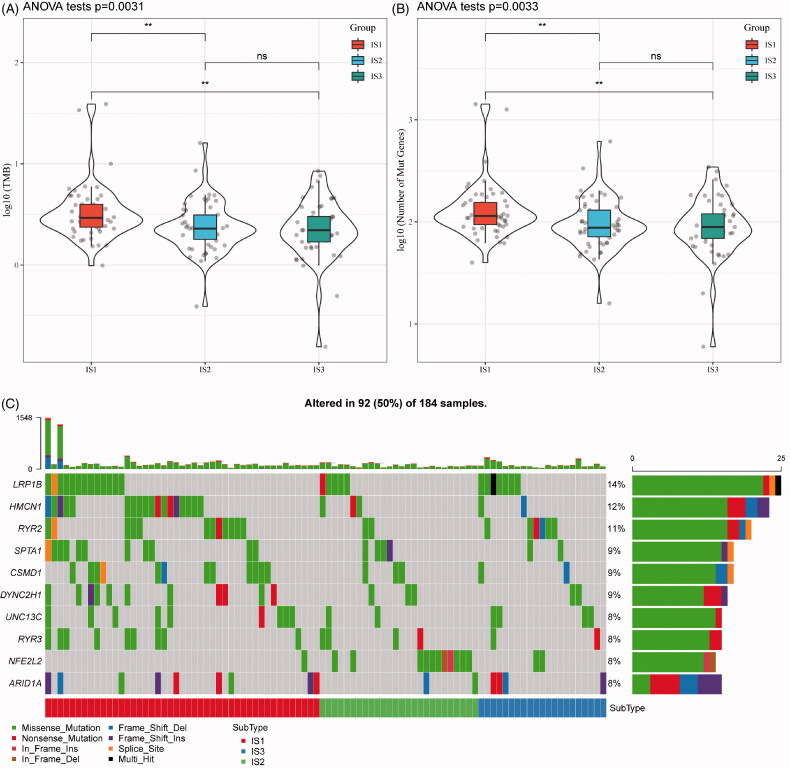
The immune subtypes are associated with TMB in oesophageal cancer. (A) The distribution of TMB in the three immune subtypes. (B) The distribution of the number of gene mutations in the three immune subtypes; the *p* value is determined by the rank sum test. (C) The characteristics of the top 10 mutation genes in each subtype.

With increasing studies supporting TMB as an independent predictive biomarker of immunotherapy, the association of TMB in the three ISs was analysed. As shown in Figure 2(A), the distribution of TMB in the three ISs revealed that TMB in IS1 subtype is significantly higher than IS2 and IS3. Moreover, the number of gene mutations in IS1 subtypes was significantly higher than that of IS1 and IS2, as shown in Figure 2(B). A total of 1278 genes with mutation in all the subtypes more than three times are listed in Table S1. Thereafter, 108 significant genes with high-frequency mutations in each subtype were screened by Chi-square test at the threshold of *p*<.05 (Table S2). The mutation features of the top 10 genes with significant mutations in each subtype were visualized (Figure 2(C)). Notably, the proportion of NFE2L2 mutations in IS2 subtypes is significantly higher than those in IS1 and IS3. A previous study found that NFE2L2 gene mutations are significantly associated with ESCC poor prognosis [21]. Intriguingly, a large proportion of ESCC patients (81.2%) was allocated to the IS2 IS.

### The features of clinical marker genes in the immune subtypes

**Figure 3.**
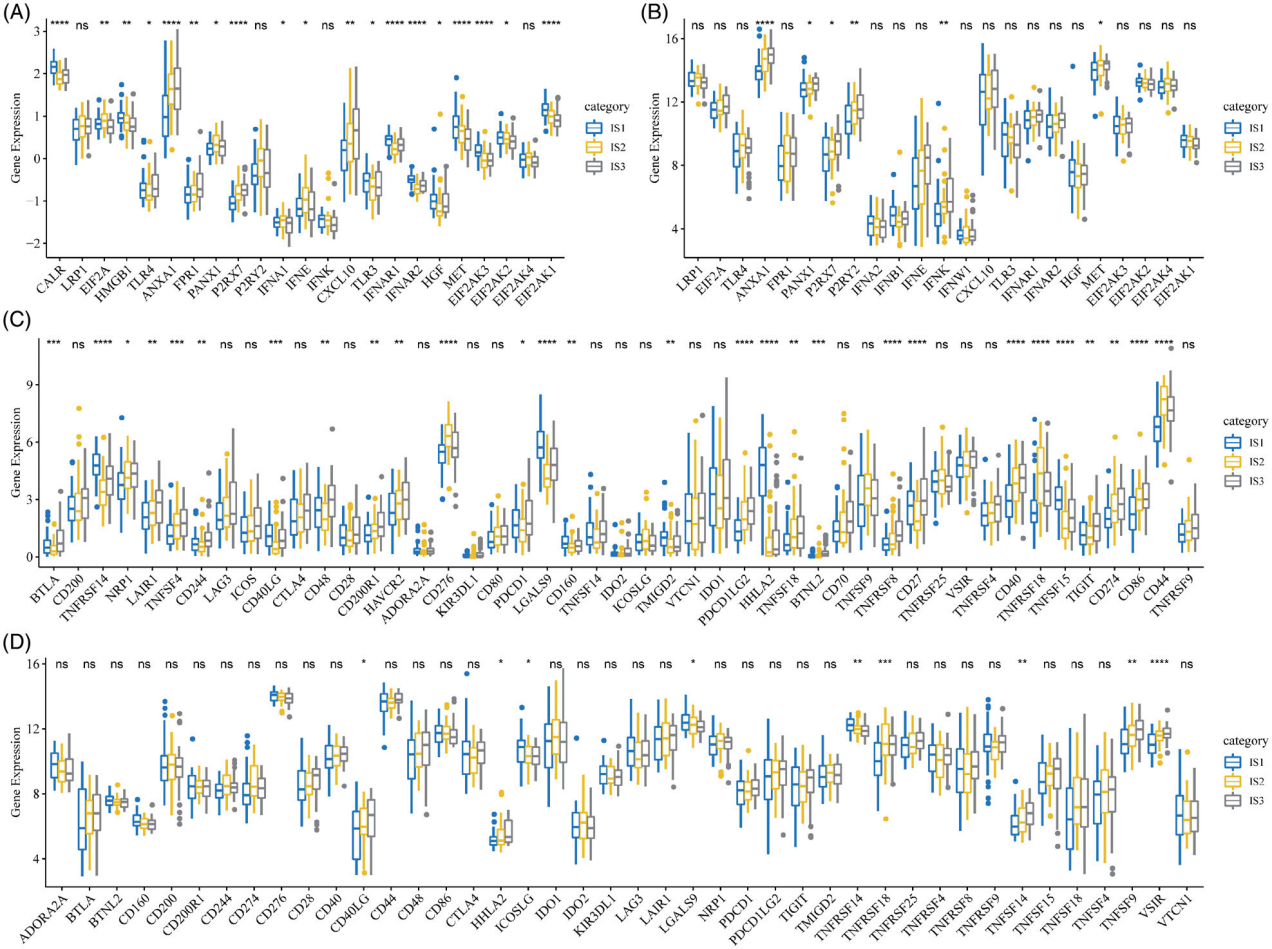
The immune subtypes are associated with immune biomarkers. (A) The expression of classic markers for chemotherapy-induced immune responses in the TCGA-ESCA cohort. (B) The expression of classic markers for chemotherapy-induced immune responses in the GSE53624 cohort. (C) Expression of immune checkpoint genes in the TCGA-ESCA cohort. (D) Expression of immune checkpoint genes in the GSE53624 cohort. The significance is statistically tested by variance analysis.

To observe the expression features of the classic markers of chemotherapy-induced immune responses in the three ISs, we retrieved these genes in the TCGA-ESCA cohort and GSE53624, respectively. In the TCGA-ESCA cohort, a total of 19 out of 23 genes (82.6%) were differentially expressed (Figure 3(A)), while six out of 23 genes in the GSE53624 cohort were differentially expressed (Figure 3(B)) in each subtype. In addition, we obtained 47 immune checkpoint-related genes from the previous study [22] and analysed the expression of these genes in our ISs. Significant differential gene expression of these genes was found in 28 (60%) in the TCGA-ESCA cohort (28/47, Figure 3(C)) and GSE53624 cohort (9/47, Figure 3(D)). Based on the TIDE algorithms, it can be observed that the IS1 subtype was more effective in immunotherapy than the other two subtypes. In subsequent gene expression analysis of immune checkpoint *PDCD1*in different ISs, the expression of PDCD1 in IS1 and IS3 was significantly higher than that of IS2 (Figure S2). These findings suggest that there are differential expression of chemotherapy-induced immune response markers and immune checkpoint-related genes in different ISs, which could be associated with clinical outcomes.

**Figure 4.**
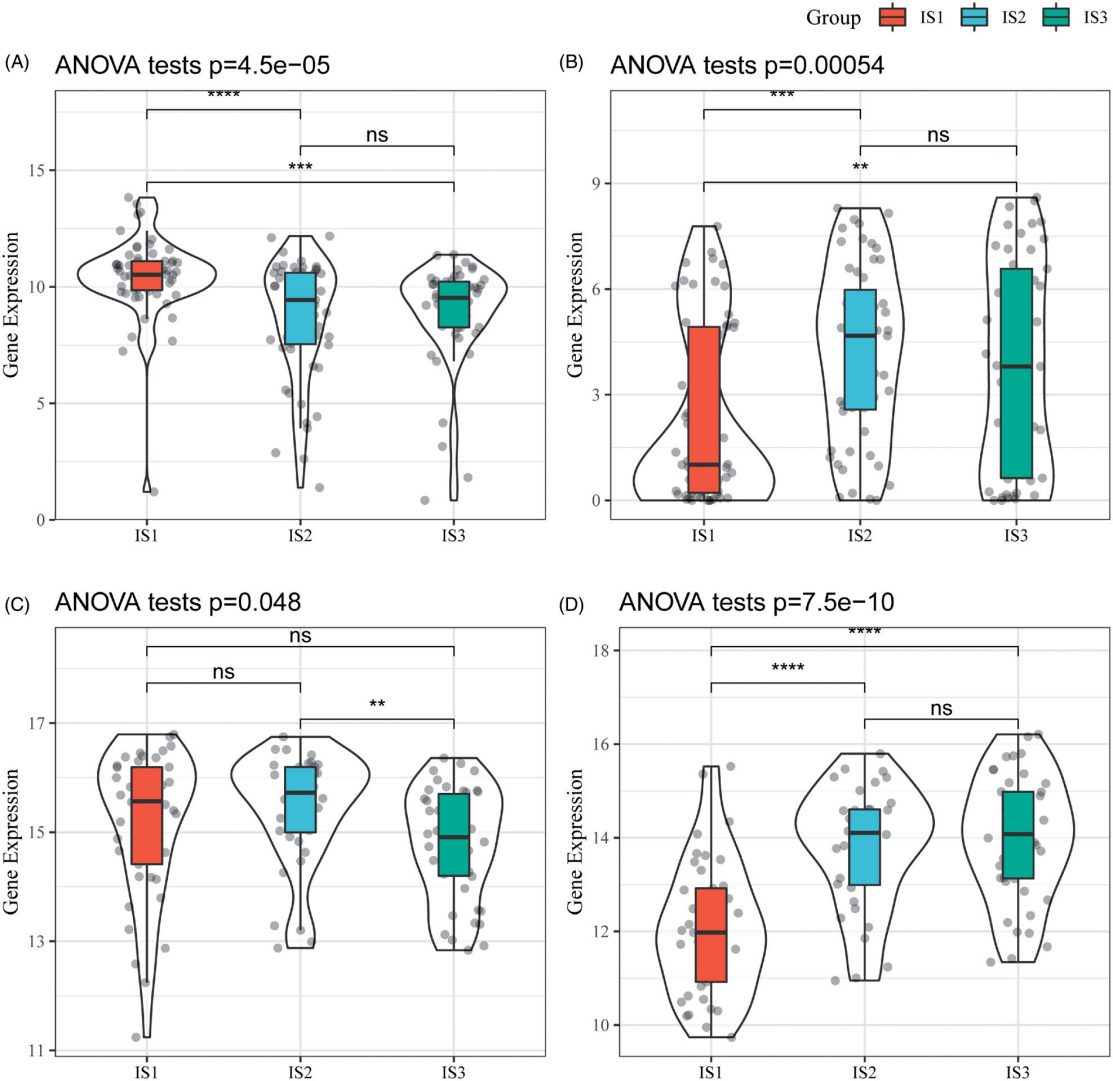
The immune subtypes are associated with biomarkers of oesophagus cancer. (A) SCC expression in each immune subtype (TCGA-ESCA). (B) Cyfra21-1 expression in each immune subtype (TCGA-ESCA). (C) SCC expression in each immune subtype (GSE53624). (D) The expression of Cyfra21-1 in each immune subtype (GSE53624).

Next, gene expression profiles of squamous cell carcinoma-associated antigen (SCC) and cytokeratin 21-1 (Cyfra21-1) from the TCGA-ESCA cohort and GSE53624 were retrieved, respectively. After assessing gene expression in each subtype, Cyfra21-1 has a good consistency in terms of differential expression between two cohorts, while the differential expression in SCC is relatively poor (Figure 4), suggesting IS1 is a more independent subtype in oesophagus cancer.

### The immune characteristics of immune subtypes and clinical implication

**Figure 5.**
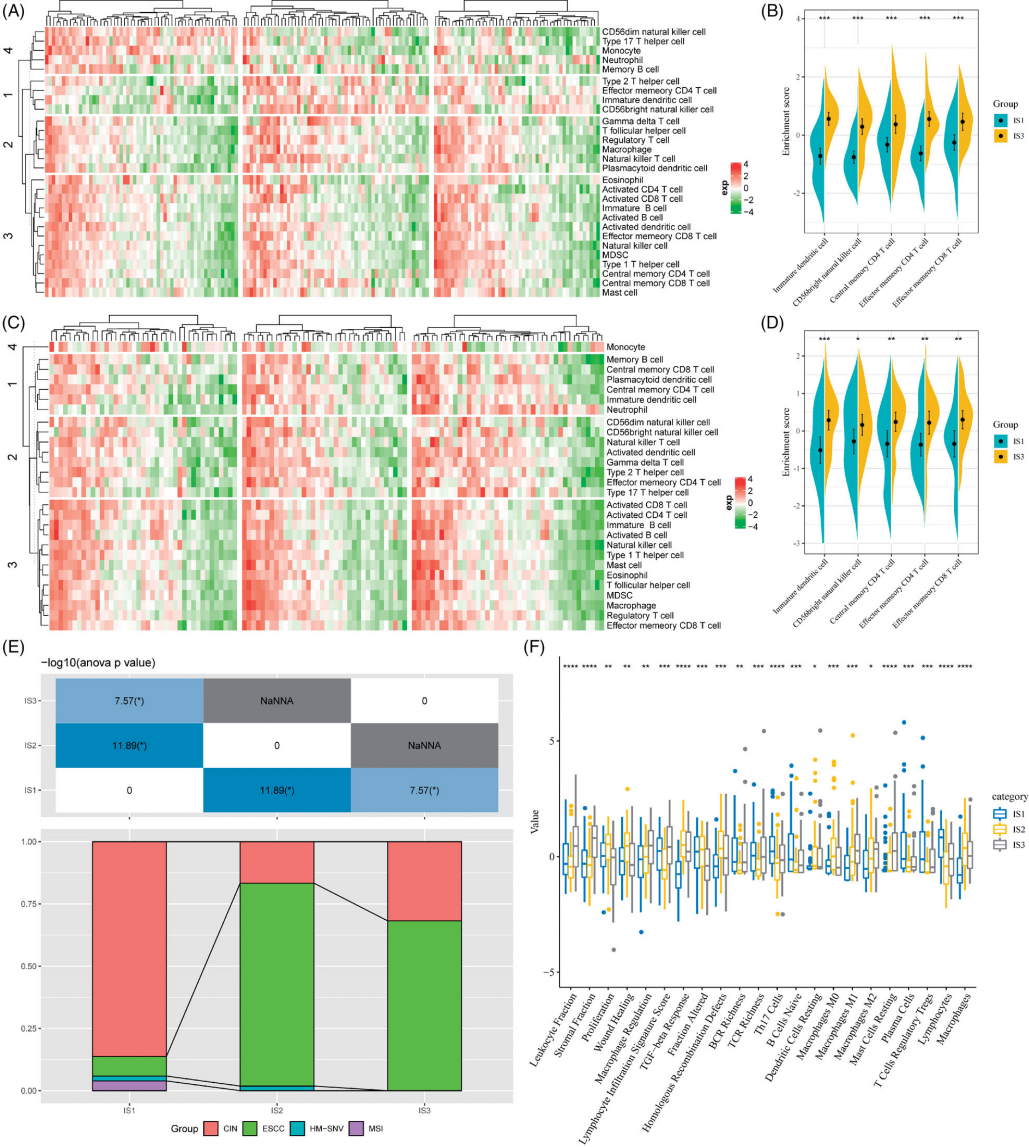
The distribution of immune subtypes in immune subpopulations. (A) The 28 immune cell enrichment score of the immune subtypes (TCGA-ESCA). (B) The enrichment score of immune cells associated with the prognosis of good and poor subtypes (TCGA-ESCA). (C) The 28 immune cell enrichment score of the immune subtypes (GSE53624). (D) The enrichment score of immune cells associated with the prognosis of good and poor subtypes (GSE53624). (E) The intersection of our three immune subtypes and previous reported molecular subtypes. (F) The distribution of three immune subtypes in 22 immune-related characteristics with significant difference (FDR < 0.05).

To study the distribution of immune cell components in the ISs, 28 immune cell marker genes from a previous study were analysed [23]. Then, the scores of 28 immune cells in each patient in the TCGA-ESCA cohort and GSE53624 cohort were determined by the single-sample GSEA method, respectively. As shown in Figure 5(A), immune cells in the TCGA-ESCA cohort were mainly divided into four categories. In addition, it can be observed that most of these immune cell components are different in each subtype, such as immature dendritic cell, CD56bright NK cell, central memory CD4 T cell, effector memory CD4 T cell, effector memory CD8 T cell were significantly lower in IS1 subtypes than IS3 subtype (Figure 5(B)). Similarly, the trends were also observed in the GSE53624 cohort (Figure 5(C,D)), which suggests that the poor prognosis of EC may be related to the inhibition of immature dendritic cell, CD56bright NK cell, central memory CD4 T cell, effector memory CD4 T cell and effector memory CD8 T cell.

To test the association of our ISs with pan-cancer molecular subtypes, the molecular subtype of EAC in previous study [15], namely chromosomal instability (CIN), hypermutation-SNV (HM-SNV) and microsatellite instability (MSI), were retrieved. In our analysis, a large proportion of EAC allocated to IS1 ISs (92.2%), while a large proportion of adenocarcinomas were classified into the CIN subtype (93.6%). Consistently, the IS1 IS and the CIN pan-cancer molecular subtypes were of the worst prognosis among respective molecular subtypes.

In addition, we also assessed the correlation of our ISs and 56 pre-defined immune molecular characteristics. With a false discovery rate of less than 0.05, 24 immune-related features were identified (Figure 5(D)). The most significant immune features of the IS1 subtype were Th17 cells, B cells naive, plasma cells, T cells regulatory Tregs and lymphocytes. Also, some immune features, such as macrophage regulation, TGF-beta response, homologous recombination defects, macrophages M1, macrophages M2, mast cells resting and macrophages were significantly higher in IS3 than in IS1 and IS2.

### The immune landscape of ESCA

**Figure 6.**
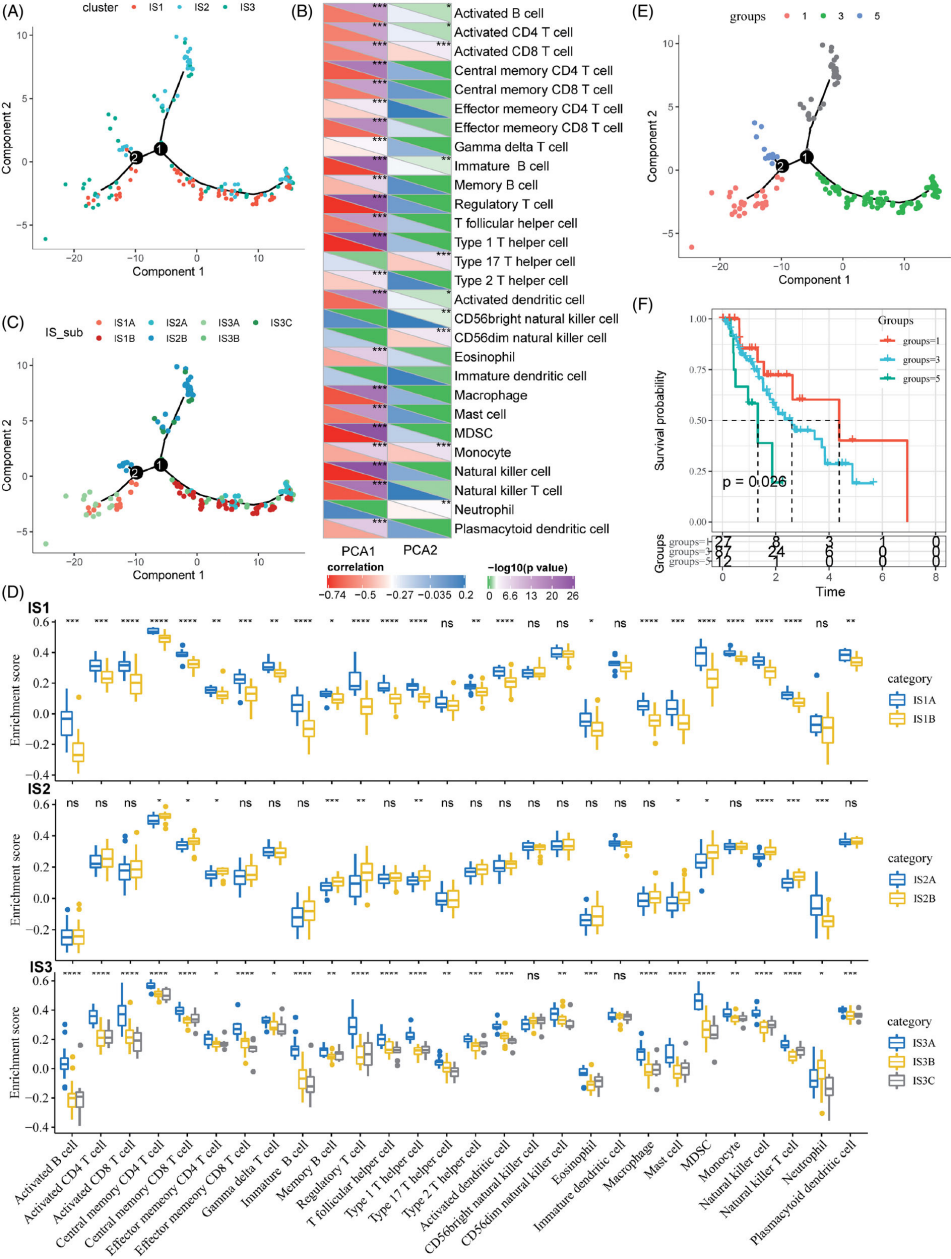
The immune landscape of oesophageal cancer. (A) The immune landscape of oesophageal cancer based on immune subtypes. (B) The correlation maps of 28 immune cell subgroups and two principal components in the immune landscape. (C) The immune landscape of oesophageal cancer based on intra-class heterogeneity of immunophenotyping. (D) The distribution of intra-class immune subtypes in immune cell subgroups. (E) The immune landscape of oesophagus based on immune subtypes with prognostic value. (F) The prognostic difference based on the locations of samples in the immune landscape of oesophageal cancer.

In this study, the immune landscape of ESCA and the overall TIME characteristics of each subtype were portraited (Figure 6(A)). Of note, the components in the horizontal coordinate were correlated with a variety of immune cells (Figure 6(B)), with the most relevant to NK cell, type 1 T helper cell, MDSC, Treg and immature B cell. The components in the vertical ordinate were related to monocyte and type 17 T helper cell. Moreover, IS1 was distributed at the opposite ends of the immune landscape, indicating a significant intra-class heterogeneity in the IS exits. According to the position of IS1 in the immune landscape map, it could be further divided into two subtypes (Figure 6(C)), with specific immune expression patterns, as shown in Figure 6(D). These results solidified the exitance of ISs with clinical value by providing different prognostic impacts of each subtype based on the immune landscape, as we defined earlier.

### Identification of key immune gene co-expression modules

**Figure 7.**
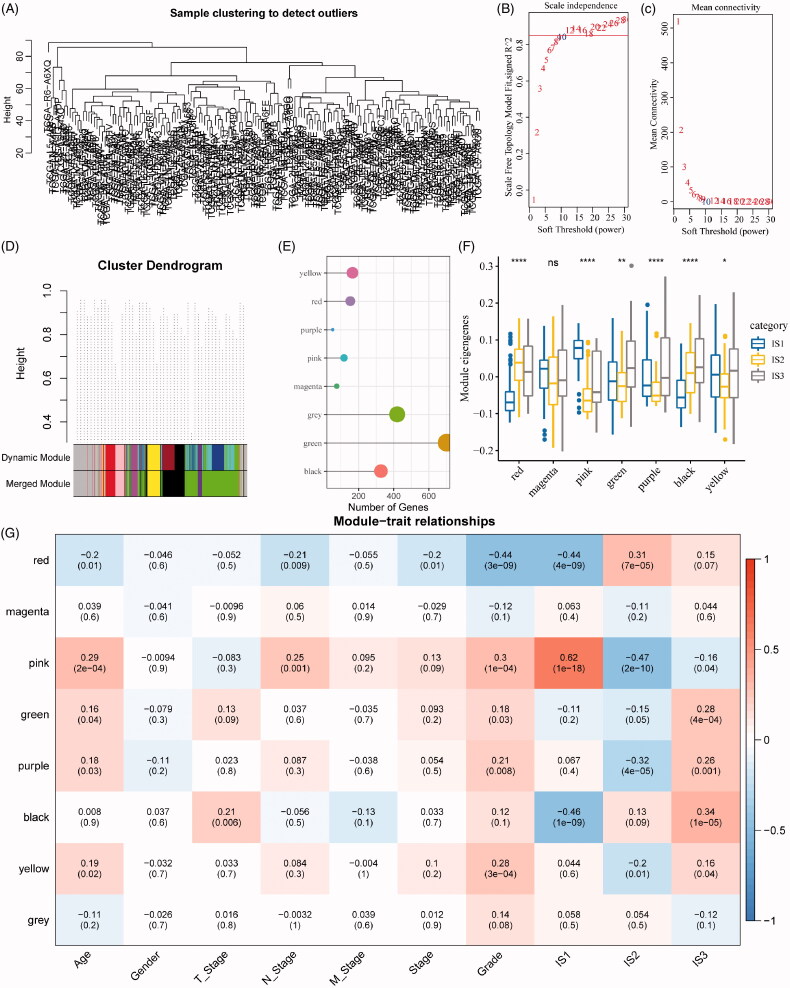
Identification of immune gene co-expression modules. (A) The cluster analysis visualized be dendrogram; analysis of network topology by scale independence (B) and mean connectivity (C). (D) The cluster dendrogram for module visualization in colours. (E) The number of gene in each module. (F) The distribution of each module in immune subtypes. (G) The correlations between co-expressed modules and clinical features as well as immune molecular subtypes.

Next, we identified the co-expression modules based on these immune genes for further understanding the biological function of our ISs. Our samples were clustered by the soft threshold of 10 in the weighted gene co-expression network analysis, as shown in Figure 7(A). A scale-free network was reached at *β* = 10 (Figure 7(B,C)). Finally, a total of seven modules were obtained (height = 0.25, deepSplit = 4, minModuleSize = 40, Figure 7(D)). As shown in Figure 7(E), it can be observed that 1951 genes were assigned to seven co-expression modules. After the analysis of the distribution of seven modules in our three immune molecular subtypes, it can be seen that some co-expression modules are differently distributed in our three molecular subtypes. In brief, the co-expression modules of IS1 in the red, green, purple, black and yellow modules are significantly lower than IS3, while those in the magenta and pink modules are significantly higher than IS3. We further analysed the correlation between each module and the patient's age, gender, T stage, N stage, M stage, stage, grade, and IS1, IS2, IS3 ISs. As shown in Figure 7(G), it can be seen that the IS1, IS2 and IS3 displayed significant correlations with the pink and black modules, respectively.

### The immune gene modules are associated with clinical traits

**Figure 8.**
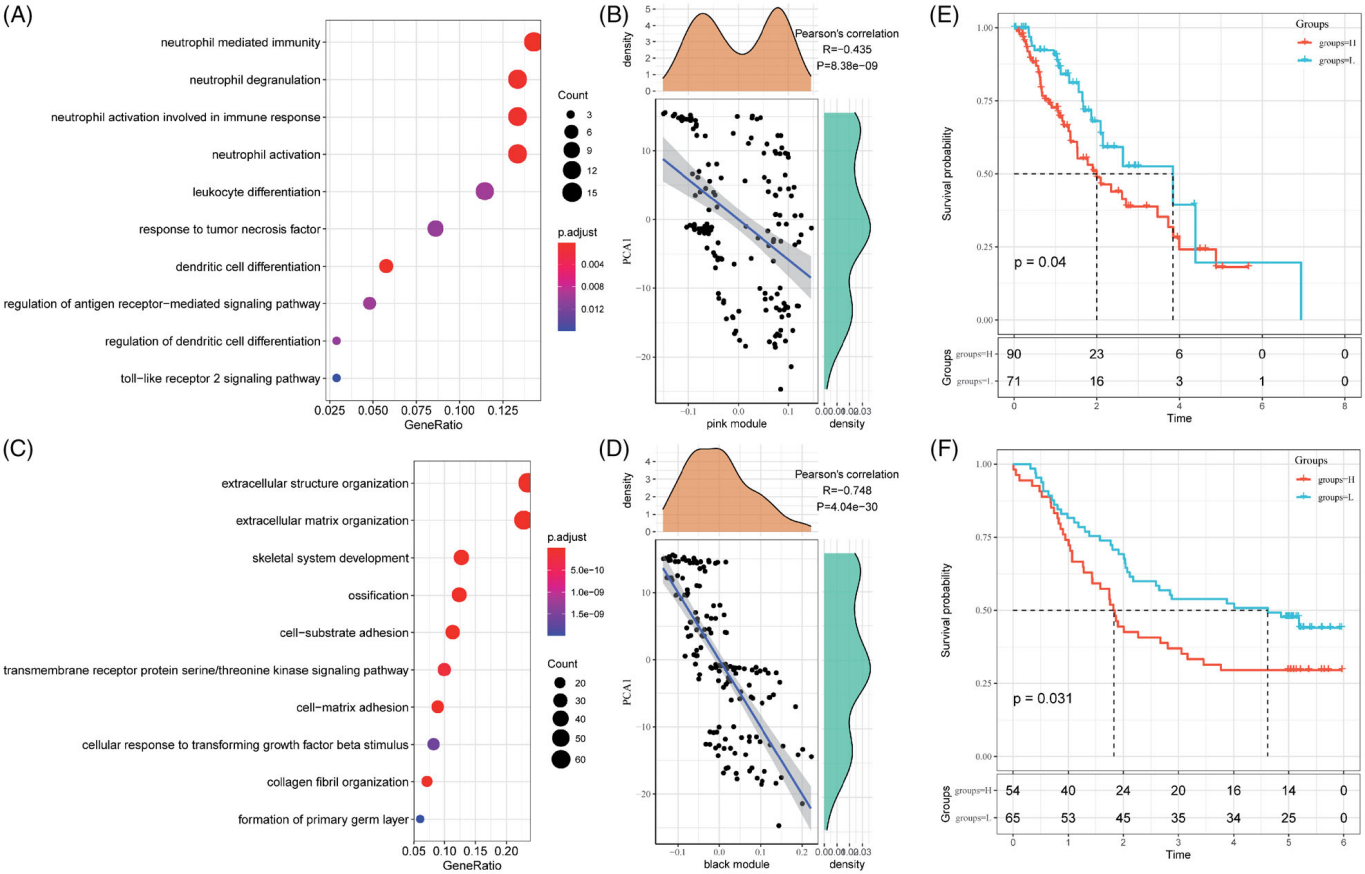
Function and prognosis analysis of immune gene co-expression module. (A) Gene enrichment analysis of pink module. (B) Correlation between the pink module and the first principal component in immune landscape. (C) Gene enrichment analysis of black module. (D) Correlation between the black module and the first principal component in immune landscape. The KM survival curve for grouping patients based on the expression of signature genes selected in the pink and black modules in TCGA-ESCA cohort (E) and GSE53624 cohort (F).

As revealing key modules in the immune cluster, gene functional analyses were performed for the investigation of their potentially affected pathways. As shown in Figure 8(A), the pink module was related to immune processes such as neutrophil activation and neutrophil activation involved in immune response, associated with the first principal component in the immune landscape (Figure 8(B)). As for the black module, an immune-mediated extracellular structure and matrix organization were indicated (Figure 8(C)), with a strong correlation with the first principal component in the immune landscape (Figure 8(D)).

For any potentially translational purpose, gene signature for prognosis prediction based on genes in these models is built. The risk score is calculated by the sum of −0.16514291 × BHLHE22– 0.03964046 × MXRA8 – 0.15242778 × SLIT2 – 0.05553572 × SPON1. As shown in Figure 8(E), the risk score can separate high-risk groups (*n* = 90) and low-risk groups (*n* = 71) in the TCGA-ESCA cohort. This model was independently validated by the clinical data in the GSE53624 cohort (Figure 8(F)).

## Discussion

The immune function in the TME is one of the major components of tumour constitution [24]. Studies pointed out that tumour-infiltrating immune cells are related to the prognosis of cancer patients [25] and therapeutic effects [26]. However, current researches mainly focussed on a single cell type [27,28]. Due to different histological types of tumours, the function of infiltrating immune cells could also be different. Even in tumours of the same pathological type, different tumour patients could have a different proportion of immune cell subgroups. Nowadays, systematic investigations on the clinically relevant TIME in EC are less reported. In this study, stable ISs in EC were classified based on immune-related genes, with independent validation, different distribution in TNM stage, and role in prognosis. In addition, the ISs of EC have different expression profile of clinical tumour markers, which are expected to evaluate the patient's tumour immune status, to understand inconsistent immunotherapy responses, and to guide combinational therapy under the dimension of TIME.

The TME contains a large number of ECM and infiltrating immune cells, but how they interact with each other is not clear. For example, cancer-associated macrophages are associated with cancer metastasis and poor prognosis. Studies have shown that TAMs can induce the epithelial–mesenchymal transition (EMT) by secretion of chemokine, thereby obtaining higher migration and invasion ability [29]. Moreover, the TAM phenotype could also form a positive feedback loop that mediates breast cancer metastasis [30]. These are evidence of how immune cells affect ECM. However, multiple molecular and cytological interactions in a tumour and even the same immune cells may perform different functions in different tumour subtypes. This study provided the IS of EC and revealed key modules functioning by immune-related genes. We noticed that the distribution of ISs in functional modules is different. It is worth noting that these functional modules act on immune-related pathways and act on various signalling pathways such as the ECM. Moreover, tumour heterogeneity may promote tumour evolution and adaptation, thus becoming a major obstacle in tumour treatment [31,32]. Considering the complex immune function, a more in-depth description of the overall characteristics of the TIME will help improve the level of individualized precision treatment. We reported for the first time that there is significant intra-class heterogeneity in ISs of EC, conferring different prognostic outcomes. Taken together, these findings alert the insufficiency of therapeutic efficacy and prognosis prediction based on a single immune index.

This study also revealed some key genes in the immune microenvironment of EC. First, four genes related to EC prognosis with potential translational significance were discovered: *BHLHE22*, *MXRA8*, *SLIT2* and *SPON1*. Interestingly, *SLIT2* expression is downregulated in EC, associating with poor prognosis [33]. A previous study showed that miR-1179 promotes cell invasion of ESCC through the *SLIT2*/*ROBO1* axis [34]. In other cancer types, *SPON1* promotes the metastasis of human osteosarcoma [35], while methylated *BHLHE22*/*CDO1*/*CELF4* panel could be used for endometrial cancer screening [36]. The association of these genes in EC worth further validation by basic and clinical studies. In addition, we found *NFE2L2* with high mutation frequency, which is associated with poor prognosis of EC [21]. How these genes predispose EC by affecting the immune microenvironment needs further study.

In summary, we propose a reproducible EC-specific IS and gene module with translational potential. We revealed the impact of ISs on molecular and cytological components in immune system as well as an intra-class heterogeneity of ISs. Therefore, a comprehensive assessment of the ISs in an individual EC sample is of great significance for understanding the personalized TIME, eventually assisting and guiding a more effective personalized immunotherapy.
